# Stereotactic ultrahypofractionated MR-guided radiotherapy for localized prostate cancer – Acute toxicity and patient-reported outcomes in the prospective, multicenter *SMILE* phase II trial

**DOI:** 10.1016/j.ctro.2024.100771

**Published:** 2024-03-25

**Authors:** C.A. Fink, J. Ristau, C. Buchele, S. Klüter, J. Liermann, P. Hoegen-Saßmannshausen, E. Sandrini, A. Lentz-Hommertgen, L. Baumann, N. Andratschke, M. Baumgartl, M. Li, M. Reiner, S. Corradini, J. Hörner-Rieber, D. Bonekamp, H.-P. Schlemmer, C. Belka, M. Guckenberger, J. Debus, S.A. Koerber

**Affiliations:** a Department of Radiation Oncology, Heidelberg University Hospital, Heidelberg, Germany; b Department of Radiation Oncology, Maria Hilf Hospital Mönchengladbach, Mönchengladbach, Germany; c Institute of Medical Biometry, Heidelberg University, Heidelberg, Germany; d Department of Radiation Oncology, University Hospital Zurich, University of Zurich, Zurich, Switzerland; e Department of Radiation Oncology, LMU University Hospital Munich, Munich, Germany; f Division of Radiology, German Cancer Research Center (DKFZ), Heidelberg, Germany; g Department of Radiation Oncology, Barmherzige Brueder Hospital Regensburg, Regensburg, Germany

**Keywords:** Prostate cancer, Stereotactic radiation therapy, MR-guided radiotherapy, Toxicity, Daily adaptive radiotherapy

## Abstract

**Background:**

Due to superior image quality and daily adaptive planning, MR-guided stereotactic body radiation therapy (MRgSBRT) has the potential to further widen the therapeutic window in radiotherapy of localized prostate cancer. This study reports on acute toxicity rates and patient-reported outcomes after MR-guided adaptive ultrahypofractionated radiotherapy for localized prostate cancer within the prospective, multicenter phase II *SMILE* trial.

**Materials and methods:**

A total of 69 patients with localized prostate cancer underwent MRgSBRT with daily online plan adaptation. Inclusion criteria comprised a tumor stage ≤ T3a, serum PSA value ≤ 20 ng/ml, ISUP Grade group ≤ 4. A dose of 37.5 Gy was prescribed to the PTV in five fractions on alternating days with an optional simultaneous boost of 40 Gy to the dominant intraprostatic lesion defined by multiparametric MRI. Acute genitourinary (GU-) and gastrointestinal (GI-) toxicity, as defined by CTCAE v. 5.0 and RTOG as well as patient-reported outcomes according to EORTC QLQ-C30 and -PR25 scores were analyzed at completion of radiotherapy, 6 and 12 weeks after radiotherapy and compared to baseline symptoms.

**Results:**

There were no toxicity-related treatment discontinuations. At the 12-week follow-up visit, no grade 3 + toxicities were reported according to CTCAE. Up until the 12-week visit, in total 16 patients (23 %) experienced a grade 2 GU or GI toxicity. Toxicity rates peaked at the end of radiation therapy and subsided within the 12-week follow-up period. At the 12-week follow-up visit, no residual grade 2 GU toxicities were reported and 1 patient (1 %) had residual grade 2 enteritic symptoms. With exception to a significant improvement in the emotional functioning score following MRgSBRT, no clinically meaningful changes in the global health status nor in relevant subscores were reported.

**Conclusion:**

Daily online-adaptive MRgSBRT for localized prostate cancer resulted in an excellent overall toxicity profile without any major negative impact on quality of life.

## Highlights

• Daily adaptive MR-guided ultrahypofractionated radiotherapy for prostate cancer is feasible with low genitourinary and gastrointestinal toxicity.
• Daily adaptive MR-guided radiotherapy has no meaningful negative impact on overall quality of life.
• Prostate-specific patient-reported outcomes remain largely unaffected after radiotherapy.

## Introduction

In patients with localized prostate cancer, stereotactic body radiation therapy (SBRT) is a standard of care option for patients eligible for radiation therapy [1], [2]. Randomized controlled trials have demonstrated that SBRT is non-inferior to conventional fractionated radiotherapy regarding biochemical recurrence free survival and acute toxicity [1], [2].

The key advantage of MR imaging is its superior soft tissue contrast. With the introduction of MR-guided radiotherapy in prostate cancer treatment, this superior contrast was combined with the ability to provide real-time visualization of pelvic anatomy and its dynamic changes. The improved visualization allows for more accurate target delineation, which in turn enables the reduction of planning target volume (PTV) margins [3] without the need for gold marker placement. Additionally, MR-guided radiotherapy offers the opportunity of online replanning to deliver an optimized plan to the patient’s daily anatomy. Moreover, the MR-guided approach allows for the observation of anatomical changes in the bladder and rectum, critical organs that can differ in volume during the course of treatment. Using beam gating, the beam is automatically turned off when the target volume moves out of its predefined localization intra-fractionally, leading to reduced radiation exposure to healthy surrounding tissues. Reduced margins, daily plan reoptimization and intrafraction-gating may further widen the therapeutic window of prostate cancer treatment, allowing for reduced treatment toxicity.

Local recurrence of localized prostate cancer after radiation typically occurs at the original dominant tumor site [4], such that dose escalation for the dominant intraprostatic lesion (DIL) carries potential for optimized local tumor control [5]. The FLAME trial demonstrates that a focal boost to the DIL improves biochemical disease-free survival in localized intermediate- and high-risk prostate cancer without adverse effects on toxicity and quality of life [6]. In recent years, dose escalation to the DIL has been safely implemented in ultrahypofractionated dose regimens [7].

The *SMILE* trial is a multicenter, prospective phase II trial aiming to evaluate safety and feasibility of online-adaptive MR-guided ultrahypofractionated stereotactic radiotherapy in prostate cancer. Here, we report on the physician-reported acute toxicity up to 12 weeks after radiotherapy and patient-reported quality of life.

## Materials and methods

### Study design and participants

The study protocol was published previously [8]. In summary, *SMILE* is a prospective, single-arm, multicenter phase II trial evaluating the feasibility and safety of ultrahypofractionated radiotherapy with online-adaptive magnetic resonance-guided radiation therapy (MRgRT) in localized prostate cancer. The inclusion criteria specified in the study protocol involved patients with low-/intermediate- and early high-grade risk groups including ≤ cT3a, ≤ Gleason Score 8, PSA ≤ 20 ng/ml, an International Prostate Symptom Score (IPSS) of ≤ 12, and a prostate gland volume of less than 80 cc. Patients with previous local therapies of the prostate were not eligible for enrolment. For risk assessment, the d'Amico criteria [9] were applied albeit T2c-tumors were categorized as intermediate risk.

In 19 out of 69 (28 %) patients with T2 tumor, there was insufficient pre-treatment data on DRE-tumor extension. Therefore, the classification as T2 relies on the baseline MRI, where no indication of extracapsular extension was observed in these patients. Patients with suspicion for nodal involvement were excluded from enrolment. Antiandrogen therapy was allowed per treating physicians’ discretion with a maximum permitted neoadjuvant treatment period of 3 months.

The study was approved by the local Institutional Review Boards of all three centers (Heidelberg University, LMU Munich, University of Zurich).

### Treatment planning and dose specifications

All patients underwent a 0.35 T MRI simulation scan at the MRIdian LINAC (ViewRay, Inc.) using a True Fast Imaging with Steady State Procession (TRUFI) sequence [10]. Diagnostic pelvic multiparametric MRI (mpMRI) was performed for contouring in addition to standard planning computer tomography without contrast. Apart from correcting for positional setup, online plan adaptation involved a daily online-MRI scan which was registered to the MRI of the base plan based on the clinical target volume (CTV) contour. After recontouring, the base plan was applied onto the anatomy of the day. If there were any dose violations in this predicted plan of either organs at risk (OAR) dose constraints or the PTV coverage, the plan was reoptimized by the medical physicist and approved by the treating radiation oncologist.

SBRT was delivered as daily online-adaptive MR-guided step-and-shoot intensity-modulated radiation therapy with target volume gating using the MRIdian LINAC (ViewRay, Inc). In low-risk cancers the target volume was defined as the prostate. The extent of additional contouring of the seminal vesicles in intermediate-risk and high-risk patients, respectively, was in accordance with the ESTRO ACROP guidelines [11]. The CTV was expanded by 3 mm isotropically to form the PTV. Per protocol, at least 95 % of the PTV should receive 95 % or more of the prescribed dose, and the maximum dose should not exceed 107 % of the prescribed dose. A prescribed dose of 37.5 Gy was delivered in 5 fractions every other day with an optional simultaneous boost up to 40 Gy to the dominant intraprostatic lesion as defined by mpMRI. A urethral PRV was formed by adding a 2 mm margin to the urethra with a dose restriction of D_0.2cc_ ≤ 37.5 Gy. No fiducial markers, rectal spacer gels, or other rectal devices were used.

### Outcomes

The primary endpoint of the *SMILE* trial is a composite measure that includes the occurrence of grade 2 or higher genitourinary (GU) or gastrointestinal (GI) toxicity scored on the Common Terminology Criteria for Adverse Events (CTCAE) version 5.0, within one year after start of radiotherapy, as well as treatment-related discontinuation of therapy. In this work, baseline symptoms and toxicity up to 12 weeks after radiotherapy were analyzed accordingly as a secondary endpoint. This endpoint was not reached if the GU or GI symptoms equivalent to a grade 2+ toxicity are already present at baseline unless the toxicity grade increased as compared to baseline. A sample size of 69 patients was calculated to show that the rate of events for the primary endpoint (genitourinary (GU) or gastrointestinal (GI) CTCAE toxicity ≥ grade 2 within one year after the initiation of radiotherapy or treatment-related discontinuation) falls below a clinically acceptable threshold of 40 %. This calculation was done with a statistical power of 80 % and a one-sided significance level of 2.5 % using an exact binomial test. Under the alternative hypothesis, an event rate of 23.8 % was assumed based on data from Bruynzeel et al. [12]. A prespecified interim analysis was conducted after 30 patients had undergone the follow-up visit after 12 weeks with no concerns regarding the continuation of the trial.

Further secondary endpoints included patient-reported quality of life (QoL) measures such as the EORTC QLQ-C30 and QLQ-PR25.

## Results

From 03/2021 to 03/2023, 69 patients were enrolled in the trial. Baseline patients’ and treatment characteristics are displayed in Table 1. There were no reported treatment discontinuations.

**Table 1:** Patients’ and treatment characteristics.

Patients [n]	69
Age, median (IQR) [years]	68 (64–75)
Karnofsky performance status, median (IQR) [%]	100 (90–100)
Gleason Score [n] 6 7a 7b 8	19 (28 %) 34 (49 %) 14 (20 %) 2 (3 %)
Low-risk [n]	8 (12 %)
Intermediate-risk [n]	58 (84 %)
High-risk [n]	3 (4 %)
iPSA, median (IQR) [ng/ml]	7.4 (5.7 – 9.2)
IPSS, median (IQR)	7 (4 – 9)
Prostate volume, median (IQR) [ml]	38 (27 – 48)
SIB to the DIL [n]	17 (25 %)
ADT use [n]	8 (12 %)

IPSS: international prostate symptom score, SIB: simultaneous integrated boost, DIL = dominant intraprostatic lesion, ADT: androgen deprivation therapy.

### Physician-reported toxicity

**Fig. 1:**
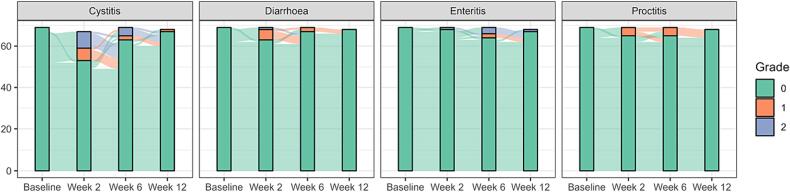
Baseline symptoms and longitudinal changes after MRgRT according to CTCAE v. 5.0 up to 12 weeks after the start of radiotherapy.

GU and GI baseline symptoms and longitudinal changes in acute toxicity according to the CTCAE definition are shown in bar plots in Fig. 1. No grade 3 toxicities were reported. During the follow-up period, 12 patients (17 %) experienced a grade 2+ GU toxicity, 6 patients (9 %) a grade 2+ GI toxicity. In total 16 patients (23 %) experienced any grade 2 GU or GI toxicity according to CTCAE up until the 12-week visit.

**Fig. 2:**
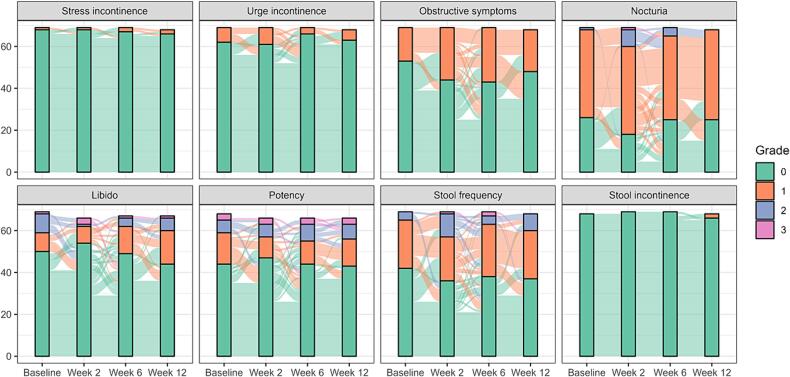
Baseline symptoms and longitudinal changes after MRgRT according to RTOG up to 12 weeks after the start of radiotherapy. Note that the toxicity endpoint in the analysis and Table 2 was not met if the GU or GI symptoms equivalent to a grade 2 + toxicity are already present at baseline unless the toxicity grade increased as compared to baseline.

GU and GI baseline symptoms and longitudinal changes of acute toxicity according to the RTOG definition [13] are shown in bar plots in Fig. 2. Apart from sexual dysfunctions, 22 patients (32 %) experienced any GU or GI grade 2+ toxicity according to RTOG up until the 12-week visit. Of note, approximately a quarter of the cohort reported baseline voiding and obstructive symptoms equivalent to a grade 1 RTOG toxicity and more than a third of patients reported nocturia equivalent to grade 1 or 2 RTOG toxicity at baseline (Fig. 2).

At the 12-week follow-up visit, only minimal residual GU and GI symptoms were reported (Table 2).

**Table 2:** Highest-grade physician-reported GU and GI toxicity up to and at the 12-week follow-up visit.

		**RTOG**			**CTCAE**		
	Highest Grade Toxicity	GU	GI	GU and/or GI	GU	GI	GU and/or GI
up to 12 weeks	I	37 (54%)	14 (20%)	32 (46%)	6 (9%)	9 (13%)	8 (12%)
	II	10 (14%)	12 (17%)	19 (28%)	12 (17%)	6 (9%)	16 (23%)
	III	1 (1%)	3 (4%)	3 (4%)	-	-	-
at 12 weeks	I	21 (31%)	8 (12%)	22 (33%)	1(1%)	-	1 (1%)
	II	-	4 (6%)	4 (6%)	-	1 (1%)	1 (1%)

### Patient-reported quality of life measures

**Fig. 3:**
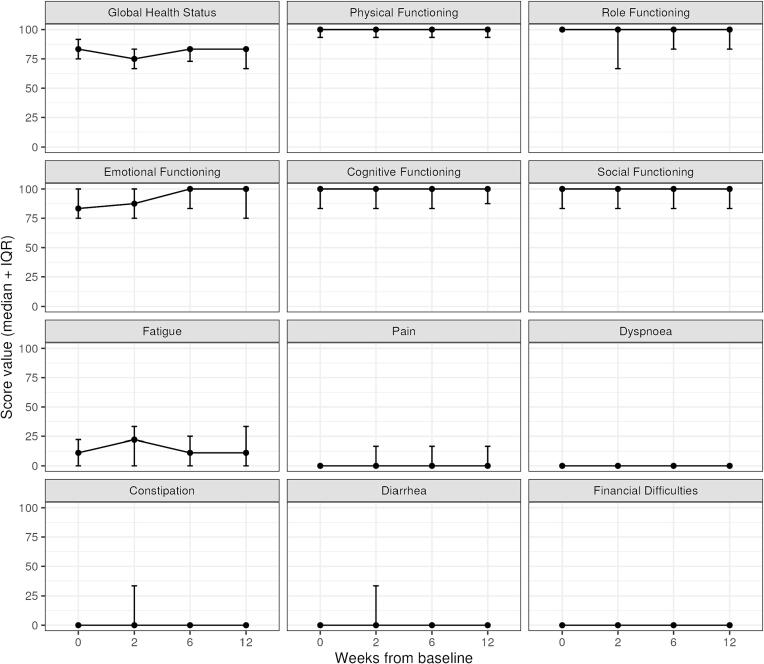
Baseline scores and longitudinal changes of QLQ-C30 scores up to 12 weeks after the start of MRgRT. Trajectories showing median and IQR.

**Fig. 4:**
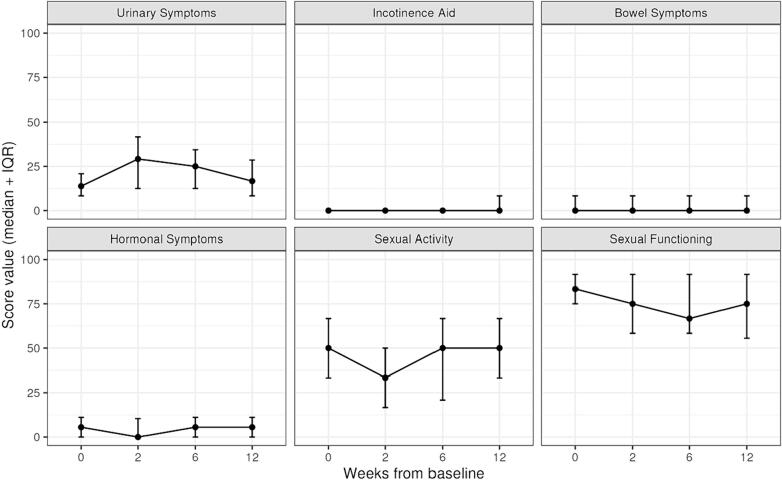
Baseline scores and longitudinal changes of QLQ-PR25 scores up to 12 weeks after start of MRgRT. Trajectories showing median and IQR.

Baseline QLQ-C30 scores and longitudinal changes after MRgRT are shown in Fig. 3. Regarding the emotional functioning subscore, there was a significant improvement at week 6 (p = 0.006) and week 12 (p = 0.039) compared to baseline as tested by the Mann-Whitney *U* test. Regarding the global health status and all relevant subscores, no clinically meaningful changes were reported. In line with physician-reported toxicity, baseline GU symptoms and sexual dysfunctions were consistent with a senior study population and most residual symptoms subsided between the follow-up visit after 6 and 12 weeks (Fig. 4).

## Discussion

The multicenter *SMILE* phase II trial aimed to demonstrate the feasibility and safety of daily online-adaptive MR-guided ultrahypofractionated stereotactic radiotherapy for localized prostate cancer. The study evaluated both physician- and patient-reported outcomes, demonstrating very favorable short-term tolerability with low toxicity rates and no clinically meaningful adverse effects on quality of life, thus supporting MR-guidance and online-adaptation in prostate SBRT.

Comparing acute toxicity profiles of previously published ultrahypofractionation regimens and their corresponding toxicity profiles is challenging due to differing dose prescriptions. The *PACE-B* trial compared acute toxicity in the context of conventionally fractionated or moderately hypofractionated radiotherapy versus five-fraction SBRT for low- to intermediate-risk localized prostate cancer. For the regimen of 36.25 Gy in 5 fractions (with 40 Gy to the CTV), RTOG GU grade 2+ toxicity was reported at 23.1 % and GI grade 2+ toxicity at 10.4 % [14]. These figures are comparable to the acute toxicity observed in the *SMILE* trial. The phase II *hypo-FLAME 2.0* trial, delivering 35 Gy in 5 fractions to the entire prostate gland with a boost up to 50 Gy to the DIL, reports GU 2+ toxicity rates of 47.5 % with biweekly radiotherapy and 34.0 % with weekly fractions [7], indicating a significant contribution of the DIL boost to the overall GU toxicity rate. In this context, an MR-guided urethral dose avoidance may mitigate acute and long-term GU toxicity in focal boost applications [15].

The introduction of MR-guidance represents a significant technical advancement in the field, offering superior soft tissue contrast and the possibility for daily online plan adaptation. As demonstrated in the randomized *MIRAGE* trial, the superior tissue contrast allows for a reduction in the PTV margin compared to CT-guided SBRT [3]. Although oncological outcomes are pending, the reported acute GU grade 2+ toxicity rates with MR-guidance were 24.4 % (compared to 43.4 % with CT-guidance) and GI grade 2+ toxicity was 0 % with MR-guidance (compared to 10.5 % with CT-guidance). While *MIRAGE* and *SMILE* are not directly comparable due to differences in radiation doses, toxicity measures, urethra constraints and the application of online plan adaptation in the *SMILE* trial, both trials report favorable GI toxicity rates in line with previously published results of trials using MRgSBRT for localized prostate cancer [12], [16]. Moreover, apart from reduced margins, MR-guidance provides the additional benefits of daily online plan adaptation and online gating. Although a recent study has indicated that gating has only a minimal impact on dose parameters [17], the use of online plan reoptimization helps to improve GTV coverage and further minimize the radiation dose to organs at risk [18], [19], [20].

Despite the favorable toxicity profile observed in this prospective multicenter trial, it is important to note its limitations, such as a relatively small patient cohort and a relatively short follow-up interval. Due to the low toxicity rates and limited patient numbers, a multivariate analysis to identify risk factors for grade 2+ toxicity development was not feasible. Nevertheless, the trial results suggest that future trials exploring markerless, ultrahypofractionated SBRT for localized prostate cancer may be designed with less stringent inclusion criteria, potentially allowing the inclusion of patients with larger prostate volumes and higher IPSS scores.

In summary, initial findings from the *SMILE* phase II trial demonstrate encouraging rates of acute gastrointestinal and genitourinary toxicity with online-adaptive MR-guided ultrahypofractionated stereotactic radiotherapy for localized prostate cancer. Patient-reported outcomes also indicate no notable impact on quality of life measures following radiotherapy. Longer-term follow-up is required to validate these early toxicity findings and support them with acceptable oncological outcome parameters.

## Funding

This study was funded by ViewRay. The industry sponsor had no role in the study design, data collection, data analysis, interpretation, or the decision to publish the findings. The content of this article reflects the independent views and research of the authors.

## Author contributions

Christoph Fink had full access to all the data in the study and takes responsibility for the integrity of the data and the accuracy of the data analysis.

Study concept and design: Körber, Klüter, Debus, Guckenberger, Belka.

Acquisition of data: Ristau, Sandrini, Buchele, Klüter, Liermann, Fink, Hoegen-Saßmannshausen, Hörner-Rieber, Andratschke, Baumgartl, Reiner, Bonekamp, Corradini, Li, Körber.

Analysis and interpretation of data: Fink, Ristau, Baumann, Körber, Schlemmer, Bonekamp, Guckenberger, Belka, Debus.

Drafting of the manuscript: Fink, Baumann, Körber.

Critical revision of the manuscript for important intellectual content: all authors.

Statistical analysis: Fink, Baumann.

Obtaining funding: Körber, Lentz-Hommertgen, Debus.

Administrative, technical, or material support: Lentz-Hommertgen.

Supervision: Körber, Andratschke, Li, Schlemmer, Bonekamp Corradini, Debus, Guckenberger, Belka.

## CRediT authorship contribution statement

**C.A. Fink:** Data curation, Writing – original draft, Writing – review & editing, Visualization, Investigation, Validation, Formal analysis, Methodology. **J. Ristau:** Writing – review & editing, Investigation. **C. Buchele:** Writing – review & editing, Investigation. **S. Klüter:** Conceptualization, Writing – review & editing, Investigation, Methodology, Supervision. **J. Liermann:** Writing – review & editing, Investigation. **P. Hoegen-Saßmannshausen:** Writing – review & editing, Investigation. **E. Sandrini:** Writing – review & editing, Investigation. **A. Lentz-Hommertgen:** Funding acquisition, Writing – review & editing. **L. Baumann:** Data curation, Writing – original draft, Writing – review & editing, Visualization, Validation, Formal analysis, Methodology. **N. Andratschke:** Writing – review & editing, Investigation, Methodology, Supervision, Resources. **M. Baumgartl:** Writing – review & editing, Investigation. **M. Li:** Writing – review & editing, Investigation, Methodology, Supervision, Resources. **M. Reiner:** Writing – review & editing, Investigation. **S. Corradini:** Writing – review & editing, Investigation, Methodology, Supervision, Resources. **J. Hörner-Rieber:** Writing – review & editing, Investigation, Resources. **D. Bonekamp:** Writing – review & editing, Investigation, Methodology, Supervision, Resources. **H.-P. Schlemmer:** Writing – review & editing, Investigation, Methodology, Supervision, Resources. **C. Belka:** Writing – review & editing, Methodology, Supervision, Resources, Project administration. **M. Guckenberger:** Conceptualization, Writing – review & editing, Methodology, Supervision, Resources, Project administration. **J. Debus:** Conceptualization, Funding acquisition, Writing – review & editing, Methodology, Supervision, Resources, Project administration. **S.A. Koerber:** Conceptualization, Funding acquisition, Writing – original draft, Writing – review & editing, Investigation, Methodology, Supervision, Resources, Project administration.

## Declaration of competing interest

All other authors declare that they have no known competing financial interests or personal relationships that could have appeared to influence the work reported in this paper.

J.H.R. received speaker fees from Pfizer Inc. and ViewRay Inc., travel reimbursement from ViewRay Inc., IntraOP Medical and Elekta Instrument AB as well as grants from IntraOP Medical and Varian Medical Systems outside the submitted work. N.A. and S.C. report honoraria from ViewRay. S.K. has received personal fees and travel reimbursement from Viewray outside the submitted work. J.D. received grants from View Ray Inc. J.D. received grants from CRI—The Clinical Research Institute GmbH, Accuray Incorporated, Accuray International Sàrl, RaySearch Laboratories AB, Vision RT limited, Astellas Pharma GmbH, Astra Zeneca GmbH, Solution Akademie GmbH, Ergomed PLC Surrey Research Park, Merck Serono GmbH, Siemens Healthcare GmbH, Quintiles GmbH, Pharmaceutical Research Associates GmbH, Boehringer Ingelheim Pharma GmbH Co, PTW-Freiburg Pychlau GmbH, Nanobiotix A.A. and IntraOP Medical outside the submitted work.
